# Comparison and Characteristics of MASLD Mouse Models

**DOI:** 10.3390/biomedicines14040895

**Published:** 2026-04-15

**Authors:** Li Wei, Chunchen Gao, Hongyan Qin

**Affiliations:** State Key Laboratory of Holistic Integrative Management of Gastrointestinal Cancers, Department of Medical Genetics and Developmental Biology, Fourth Military Medical University, Xi’an 710032, China; wlweili1202@163.com (L.W.); spring9226@163.com (C.G.)

**Keywords:** metabolic dysfunction-associated steatotic liver disease, metabolic dysfunction-associated steatohepatitis, mouse model

## Abstract

Metabolic dysfunction-associated (non-alcoholic) steatotic liver disease (MASLD) is a chronic inflammatory liver disorder characterized by excessive hepatic lipid accumulation. Its progressive subtype, metabolic dysfunction-associated (non-alcoholic) steatohepatitis (MASH), is featured by enhanced inflammation and liver injury. Some MASH cases are accompanied by hepatic fibrosis, which may progress to cirrhosis and hepatocellular carcinoma (HCC). MASLD is also associated with comorbidities such as cardiovascular disease and chronic kidney disease. To date, only Resmetirom has been approved by the FDA for MASH treatment, highlighting the urgency of investigating MASH pathogenesis and developing effective therapeutic agents. Establishment of experimental animal models which can mimic the clinical symptom of MASLD are fundamental to explore therapeutic targets and advance clinical drugs development. Therefore, this review focus on the pathological features of MASLD/MASH and comprehensively summarizes the current MASH-related mouse models, which can be useful for researchers to select appropriate models in order to explore the underlying mechanisms and dig novel targets for MASH treatment.

## 1. Introduction

The definition of metabolic dysfunction-associated steatotic liver disease (MASLD) was officially proposed in 2023, replacing the established diagnostic criteria for non-alcoholic fatty liver disease (NAFLD) [1]. This diagnostic criterion applies to patients presenting with hepatic steatosis alongside at least one of the five components of metabolic syndrome. Relevant metabolic risk factors include abnormal body mass index (BMI), waist circumference, blood glucose, blood pressure, triglycerides, and cholesterol levels [2]. As a major chronic metabolic syndrome, MASLD affects over 38% of the global adult population and has emerged as a significant public health concern [3,4,5]. Metabolic dysfunction-associated steatohepatitis (MASH), a progressive form of MASLD, is characterized by severe inflammation and liver injury, potentially leading to advanced fibrosis, cirrhosis, and hepatocellular carcinoma (HCC). Currently, liver biopsy remains the sole method for definitive MASH diagnosis. The rising prevalence of MASH has increased the global incidence and mortality of liver diseases and has become a primary indication for liver transplantation [6]. In Asia, MASH has surpassed hepatitis B as the leading cause of chronic liver disease. Notably, 35% of hepatitis B patients also have MASLD, a comorbidity that further exacerbates liver damage [7].

Recent investigations into the molecular mechanisms and therapeutic strategies of Metabolic Dysfunction-Associated Steatotic Liver Disease (MASLD) have significantly advanced our understanding of its epidemiology, pathogenesis, and potential therapeutic targets. Notably, the recent FDA approval of Resmetirom for adults with MASH and liver fibrosis marks a pivotal breakthrough in the field [8]. However, the high heterogeneity of MASLD necessitates ongoing research into alternative treatment strategies. For instance, researchers are focusing on identifying stratified biomarkers to optimize patient-specific therapeutic approaches, particularly immunotherapies for MASLD-related HCC [9].

The heterogeneity of human MASLD is influenced by genetic susceptibility, dietary patterns, and gut microbiome composition. Consequently, establishing preclinical animal models that recapitulate this heterogeneity is imperative. Mice share approximately 85% genetic homology with humans, making them an ideal model organism for elucidating human disease mechanisms and evaluating therapeutic interventions. This review systematically describes established and emerging MASLD/MASH mouse models, providing a detailed explanation of how dietary, genetic, and chemical induction methods can reproduce specific disease stages across the MASLD spectrum. By offering key insights into model selection and application, this review aims to support researchers in conducting mechanistic studies and advancing clinical therapies for this increasingly prevalent metabolic liver disease.

## 2. Core Pathogenic Mechanisms of MASLD/MASH

MASLD is a complex, multifactorial chronic liver condition driven by a sophisticated interplay of genetic and environmental factors. The pathogenesis is underpinned by core mechanisms including insulin resistance (IR), genetic susceptibility, environmental triggers, dysbiosis of the gut microbiota, and immune-inflammatory responses.

### 2.1. IR: Central Node of Lipid Metabolism Disorder

IR acts as the primary driver and central mechanism in the pathogenesis of MASLD [10]. Under conditions of IR, the anti-insulin effect in adipose tissue is amplified, accelerating lipolysis and the subsequent release of substantial free fatty acids (FFAs) into the circulation for hepatic uptake [11]. Concurrently, hepatic sensitivity to insulin signaling diminishes; while the suppression of gluconeogenesis is attenuated, the stimulation of lipogenesis exhibits selective preservation. This leads to a marked upregulation of de novo lipogenesis (DNL) pathways, characterized by heightened activity of key transcription factors, including SREBP-1c and ChREBP, which drive the expression of critical enzymes such as fatty acid synthase (FAS), ultimately resulting in massive triglyceride accumulation within hepatocytes [12]. This lipid accumulation is inherently lipotoxic, further aggravating hepatic IR and endoplasmic reticulum stress, thereby establishing a self-perpetuating vicious cycle.

### 2.2. Genetic Susceptibility and Epigenetic Regulation

MASLD exhibits significant familial aggregation and a strong genetic predisposition. Genome-wide association studies (GWAS) have identified several key susceptibility loci. Notably, the I148M variant in the patatin-like phospholipase domain containing 3 (*PNPLA3*) gene is currently recognized as the most robust genetic risk factor, significantly increasing hepatic fat content as well as the risks of inflammation and fibrosis [13]. Furthermore, the E167K variant in the *TM6SF2* gene is closely associated with hepatic lipid accumulation and disease progression [14]. Additionally, polymorphisms in *GCKR* and *MBOAT7* contribute to MASLD susceptibility. Epigenetic modifications also play a crucial role; alterations in DNA methylation, histone modifications, and non-coding RNAs (e.g., miR-122) influence disease phenotypes by regulating the expression of genes involved in lipid metabolism and inflammation.

### 2.3. Environmental Exposure, Microbiota, and Immune Inflammation

Unhealthy lifestyle factors, including high-fat diets and sedentary behavior, represent the principal environmental drivers of MASLD. Furthermore, gut microbiota dysbiosis is pivotal in modulating “gut–liver axis” communication. Impaired intestinal barrier function increases permeability, facilitating the translocation of microbial products, such as lipopolysaccharide (LPS), into the portal circulation and resulting in “endotoxemia” [15]. LPS activates Toll-like receptor 4 (TLR4) on hepatic Kupffer cells (KCs), triggering downstream inflammatory signaling pathways and the release of substantial pro-inflammatory cytokines, including tumor necrosis factor-α (TNF-α) and interleukin-1β (IL-1β). This inflammatory milieu is a critical factor driving the progression from simple steatosis to MASH [16].

Persistent hepatocyte injury and inflammatory responses recruit and activate various immune cells. Specifically, hepatic macrophages, neutrophils, and T cell subsets release pro-fibrotic mediators, such as transforming growth factor-β (TGF-β), which directly activate hepatic stellate cells (HSCs) [17]. Activated HSCs serve as the primary effectors of hepatic fibrosis; they proliferate extensively and secrete extracellular matrix components, ultimately leading to fibrosis and potentially cirrhosis. Genetic susceptibility and environmental factors synergistically shape gut microbiota composition, collectively accelerating the pathological progression of MASLD [18].

## 3. Classification and Construction Strategy of MASLD Animal Models

**Figure 1:**
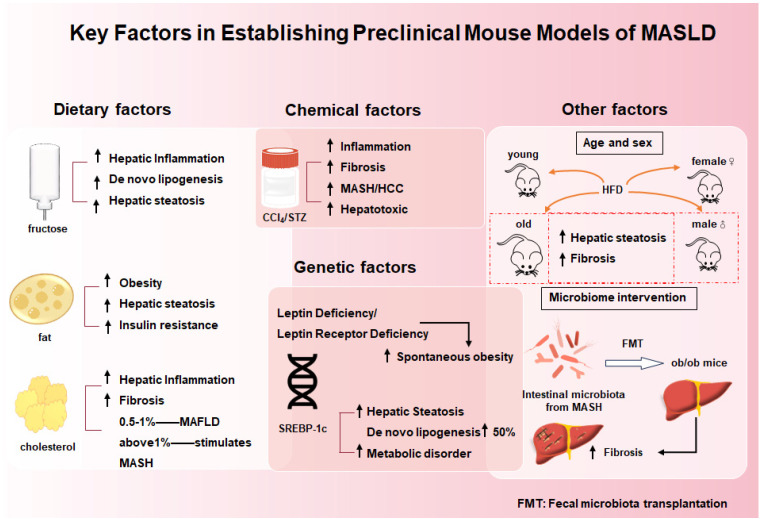
Key factors and pathways in establishing preclinical mouse models of MASLD. The schematic illustrates the primary methodologies for inducing MASLD in mice, categorized into dietary, chemical, and other factors (including genetic and microbiome interventions). These factors contribute to the disease spectrum through interconnected pathways leading to hepatic steatosis, inflammation, fibrosis, and progression to MASH and HCC. Key pathological processes such as enhanced DNL (contributing up to 50% of hepatic lipid content) and critical thresholds for disease progression (e.g., hepatic steatosis > 0.5–1%) are highlighted. FMT, fecal microbiota transplantation; STZ, streptozotocin. Arrows indicate increase.

Developing robust preclinical animal models is crucial for enhancing the success rate of clinical trials and evaluating the efficacy of therapeutic drugs prior to clinical enrollment. Therefore, this review focuses on preclinical animal models for MASLD, with particular emphasis on their ability to effectively mimic the human disease. Considering that environmental factors and genetic susceptibility are driving the pathogenesis of MASLD/MASH, preclinical mouse models are currently categorized into three groups: diet-induced, genetically engineered, and toxin/drug-induced models (Figure 1). In the following sections, we will discuss the complex mechanisms underlying these different MASLD/MASH mouse models and summarize their respective advantages and limitations for preclinical research. These insights aim to provide a comprehensive reference for future model selection.

### 3.1. Diet-Induced MASLD Mouse Model

Intracytoplasmic lipid accumulation in the form of triglycerides is key character of MASLD. Hepatic lipid synthesis generally involves two primary pathways: the uptake of FFAs from circulation and DNL. Triglycerides and cholesterol stored within hepatocyte lipid droplets are metabolized to release FFAs via lipolytic and lysosomal hydrolysis pathways. These FFAs are subsequently utilized by hepatocytes through peroxisomal and mitochondrial fatty acid β-oxidation pathways, providing metabolic fuel for other tissues and serving as a lipid precursor for very low-density lipoprotein (vLDL) synthesis. However, under metabolic stress, dietary sugars (particularly fructose) promote hepatic lipid accumulation [19]. In the context of IR, increased dietary uptake and the simultaneous release of FFAs from adipocytes lead to elevated hepatic FFA import [20,21]. When the influx of FFAs exceeds the hepatic metabolic capacity, hepatocellular triglyceride reservoirs expand. Consequently, FFA metabolites such as ROS and ceramide induce hepatocellular damage and ballooning, leading to steatohepatitis [22].

Diet-induced models are based on the pathogenesis of overnutrition, characterized by the excessive intake of lipids, cholesterol, or sugars [23]. Conversely, lobular and portal inflammation are prominent features of MASLD resulting from the dysregulation of cytokine and chemokine expression. Therefore, hepatic lipid accumulation and subsequent inflammatory responses are considered major contributors to the development of MASLD. Representative mouse models reflecting these two key features are described below (Table 1).

#### 3.1.1. High-Fat Diet (HFD)-Induced Mouse Models

High-fat diet (HFD)-fed mice are widely utilized to study hyperlipidemia, hyperglycemia, and MASLD. Specifically, specific-pathogen-free (SPF) grade C57BL/6J mice are frequently employed to establish MASLD models via high-glucose/high-fat diet feeding. This model recapitulates key features of the human disease, including obesity, IR, and lipid metabolism disorders.

The nutritional composition of HFD formulations varies, with common fat content ranging from 40 to 60% or from 45 to 75% [24]. Feeding mice a 45% fat diet for 6–12 weeks induces hepatic lipid accumulation and mild IR, generating a simple steatosis model that reproduces early features of human MASLD. Extending the feeding period to 16 weeks with 45% or 60% fat diets promotes obesity, hepatic steatosis, and IR, simulating the transition from simple steatosis to steatohepatitis in humans [25]. Notably, 24 weeks of HFD feeding causes significant liver inflammation and early fibrosis, reflecting early human steatohepatitis. Similarly, administering a HFD (such as 71% fat, 11% carbohydrate, and 18% protein) for 16 weeks induces IR accompanied by significant lobular steatosis, inflammation, and fibrosis [26], thereby recapitulating early human steatohepatitis. In the progression of MASLD, the gut microbiota significantly impacts liver lipid metabolism, independent of obesity [27]. One week of HFD feeding is sufficient to induce gut microbiota imbalance and bacterial translocation into the liver, further exacerbating MASLD [28].

Although the HFD feeding model is valuable for exploring the typical characteristics of MASLD, the complete pathological spectrum of human steatohepatitis, particularly in the late stages, has not been fully recapitulated in mice. To overcome this limitation, researchers have developed combinatorial models that incorporate genetic or chemical interventions, enabling a better simulation of human steatohepatitis progression (see below).

#### 3.1.2. Methionine- and Choline-Deficient (MCD) Diet-Induced Mouse Model

The MCD diet contains 40% sucrose and 10% fat, lacking methionine—an essential amino acid critical for protein synthesis—and choline, which facilitates liver lipid transport via phospholipid synthesis and prevents abnormal lipid accumulation. Mechanistically, deficiencies in methionine and choline impair mitochondrial β-oxidation and vLDL synthesis, thereby disrupting intrahepatic triglyceride export [29,30]. This diet rapidly induces key features of MASLD in mice within 2–4 weeks, including steatosis and hepatocellular ballooning. During this process, upregulation of cyclooxygenase-2 (COX-2) and monocyte chemoattractant protein-1 (MCP-1) promotes neutrophil and monocyte infiltration, leading to hepatic inflammation and fibrogenesis [31].

The MCD model effectively reproduces early human MASLD phenotypes, particularly hepatic steatosis. After 8 weeks of feeding, mice develop significant liver fibrosis, simulating mid-stage steatohepatitis. Extending the dietary intervention to 12–18 weeks can lead to malignant tumor progression, mimicking the clinical transition from human cirrhosis to HCC [32].

In addition to hepatic injury, the MCD diet impairs intestinal barrier integrity and alters gut microbiota composition and metabolites; however, these microbial shifts are less representative of human MASLD than those observed in the high-fat, high-cholesterol (HFHC) model [33,34,35].

Notably, unlike many other MASLD models, MCD model does not induce IR despite disturbing glucose metabolism. It primarily enhances fatty acid uptake and inhibits the synthesis and secretion of vLDL, disrupting liver lipid homeostasis and leading to pronounced fibrosis in a short period of time. Notably, transcriptome analysis showed limited transcript overlap between MCD model and human MASH, despite changes in fibrosis-related genes, significant differences were observed in key events such as metabolism-related pathways, particularly DNL, and PNPLA3 driven disease-associated signaling pathways [36]. Moreover, MCD-fed mice exhibit decreased body and liver weights, which contrasts with the weight gain typically observed in human MASLD patients.

Collectively, the HFD and MCD diets are the two most widely used dietary models for studying MASLD, each exhibiting distinct molecular and pathological phenotypes. Consistent with previous reports, our comparative analysis revealed distinct kinetics of hepatic macrophage infiltration between these models. In the MCD model, hepatic F4/80+ macrophage accumulation is markedly enhanced within a short timeframe, reflecting a robust and rapid induction of innate immune inflammation associated with steatohepatitis. In contrast, the HFD model exhibits a more gradual, time-dependent increase in hepatic macrophage content, with significant infiltration observed only after prolonged feeding (e.g., 24 weeks), mirroring the chronic inflammatory nature of human MASLD pathogenesis.

#### 3.1.3. HFHC Diet-Induced Mouse Model

Cholesterol accumulation in the liver is a critical risk factor for the development of MASH. The high-cholesterol diet (HCD) is typically low in fat and contains approximately 1% cholesterol and 4% fat [37,38]. While HCD-fed mice exhibit elevated hepatic cholesterol ester and serum ALT levels, they do not typically develop full steatohepatitis. A critical limitation of the HCD model is the induction of supraphysiological cholesterol levels in vivo. To address this limitation, researchers developed the HFHC diet. The synergistic effects of dietary fat and cholesterol in the HFHC model induce pathological features of human MASLD, circumventing the hypercholesterolemia disadvantage observed in the HCD model.

The HFHC diet generally contains >15% fat and 0.2–1% cholesterol, frequently supplemented with sucrose and cholate. The disease phenotype in HFHC-fed mice develops in a time-dependent manner, closely mirroring the clinical stages of human MASLD. Feeding mice this diet for 8–12 weeks results in obesity and hepatic steatosis, accompanied by elevated plasma ALT and reduced adiponectin levels, effectively simulating early MASH progression. After 16–24 weeks, inflammatory infiltration and mild fibrosis emerge, reproducing characteristic features of human MASH. Notably, a specific formulation containing 30% fat, 1.25% cholesterol, and 0.5% bile acid induces hepatic ballooning within 16–24 weeks [39]. During this process, dysregulated bile acid metabolism acts as a core driver, directly promoting liver injury and inflammation while activating HSCs to drive fibrosis [40]. Extending the feeding duration to over 36 weeks exacerbates fibrosis, simulating the late stages of human MASLD [26].

Collectively, the HFHC diet induces a simple and reproducible MASH model that is widely utilized to study disease mechanisms and evaluate potential treatments. However, its application is somewhat limited by inconsistent effects on insulin resistance, making it more suitable for diet-induced obesity (DIO)-susceptible strains, such as C57BL/6 mice. To enhance the clinical translatability of HFHC-induced MASH, common strategies include prolonging the high-fat diet feeding period, introducing genetic modifications, or administering secondary harmful stimuli to exacerbate disease phenotypes.

#### 3.1.4. Choline-Deficient L-Amino-Defined Diet (CDAA)-Induced Mouse Model

Compared with mice fed a MCD diet, those fed a CDAA diet overcome the disadvantage of weight loss and do not exhibit IR. Following 3 weeks of dietary intervention, CDAA-fed mice show significantly elevated serum TNF-α levels, upregulated TLR4 and CD14 expression, and histopathological features of MASLD, including hepatocyte lipid deposition, inflammatory infiltration, and elevated serum ALT levels [41], These characteristics accurately simulate the transition from simple steatosis to early MASH in humans.

Supplementing the CDAA diet with high fat accelerates MASLD development, exhibiting severe steatosis and remarkable inflammatory responses within 6 weeks, followed by fibrosis. These features are consistent with human MASH with an early fibrotic phenotype [42,43]. Upon extending the feeding period to 36 weeks, the CDAA-HFD model progresses to hepatocellular adenoma (HCA) and HCC, effectively recapitulating the clinical process from chronic steatohepatitis to liver cancer.

Notably, the CDAA-HFD typically contains 0.1% methionine and 45% fat [44]. However, another study reports that a CDAA-HFD with 60% fat and 0.1% methionine induces hepatic fibrosis in mice after 6 weeks of feeding [42]. In particular, one-week of CDAA-HFD feeding in mice induces fatty liver and mitochondrial dysfunction, accompanied by severe liver oxidative stress but without fibrosis, effectively mimicking the early stages of human MASH [45].

#### 3.1.5. Gubra-Amylin NASH (GAN) Diet-Induced Obese (DIO) Mouse Model

The Gubra-Amylin NASH (GAN) diet-induced obese mouse model (GAN DIO-MASH) reproduces classical metabolic and histopathological features of human MASH, including progressive liver fibrosis. Feeding *C57BL/6J* mice a specialized formula diet (40% saturated fat, 22% fructose, and 2% cholesterol) for more than 38 weeks induces a phenotype characterized by obesity, hypercholesterolemia, hyperinsulinemia, and impaired glucose tolerance, accompanied by steatosis, inflammation, and hepatocyte ballooning [46].

Recent studies have shown that the GAN feeding duration can be shortened in aged mice. Fifty-six-week-old mice develop steatosis, lobular inflammation, and ballooning after 12 weeks of GAN diet feeding, whereas extending the duration to 21 weeks induces severe fibrosis, resulting in a more advanced MASH phenotype compared to young (5-week-old) counterparts [47]. Consequently, the accelerated progression of MASH due to aging significantly shortens the experimental timeline for preclinical research.

In addition to histopathological consistency, the GAN DIO-MASH model exhibits high clinical relevance at the transcriptome level, as evidenced by liver gene expression profiles similar to those of human MASH samples [48]. Given its comprehensive recapitulation of human MASH characteristics—including metabolic disorders, histopathological features, and molecular signatures—this model serves as an ideal translational tool for exploring MASH pathogenesis, identifying therapeutic targets, and evaluating drug efficacy.

#### 3.1.6. “American Lifestyle-Induced Obesity Syndrome” (ALIOS) Mouse Model

The ALIOS mouse model is a highly suitable platform for investigating MASLD progression, particularly as it accurately mimics human MASLD induced by a Western diet rich in trans fats and fructose. This model involves feeding mice a high-fat diet (containing 30% trans fatty acids) and administering high-fructose corn syrup (containing 55% fructose and 45% sucrose) in drinking water, effectively inducing obesity, insulin resistance, and hepatic steatosis accompanied by necroinflammatory changes. Long-term feeding for up to 12 months further promotes the progression of MASH and increases susceptibility to HCC [49].

A defining feature of the ALIOS model is its distinct metabolic and hepatic pathological characteristics. Metabolomics analysis following 8–16 weeks of WD diet feeding—representing the early to mid-stages of MASLD—reveals that dietary intervention induces progressive alterations in hepatic lipid composition. These changes are marked by a decrease in lipids containing polyunsaturated fatty acids, an increase in other lipid species, and a reduction in both antioxidant metabolites and gut microbiota-derived metabolites. Collectively, these metabolic shifts contribute to MASLD progression [50].

Currently, clinical management of MASLD primarily focuses on improving insulin sensitivity through lifestyle modifications, pharmacotherapy, or bariatric surgery; however, current therapeutic options remain limited. By recapitulating the key dietary and metabolic drivers of human MASLD, the integration of untargeted metabolomics with transcriptomic profiling in the ALIOS model has emerged as a robust strategy for identifying novel, pathway-targeted therapeutic interventions [50].

### 3.2. Chemical Compound and High-Fat-Induced Composite Model

Clinical evidence indicates that 41% of MASH patients may develop progressive liver fibrosis, with 25% of these individuals subsequently progressing to cirrhosis [51]. Administration of carbon tetrachloride (CCL4) alone induces liver fibrosis without concomitant obesity or IR. However, combining CCL4 administration with a HFD feeding promotes the onset of MASH and accelerates hepatic fibrosis [52,53]. Furthermore, while streptozotocin (STZ) injection is typically used to induce type 2 diabetes models, a low-dose STZ regimen administered neonatally, followed by HFD feeding, can induce steatohepatitis, fibrosis, and even HCC [54] (Table 2).

#### 3.2.1. High-Fat Diet Combines with Low-Dose CCL4-Induced MASH Model

During the early phase (2–4 weeks) of CCl_4_ administration combined with HFD feeding, low-dose CCl_4_ (0.5–0.8 mL/kg) induces hepatocyte injury and initial fibrotic responses, characterized by hepatic stellate cell activation and nascent collagen deposition [55]. As the intervention progresses to the intermediate stage (8–12 weeks), the synergistic effects of dietary metabolic stress and recurrent chemical injury promote severe MASH fibrosis, marked by hepatocyte ballooning, lobular inflammation, and progressive extracellular matrix remodeling. Upon reaching the late stages (16–24 weeks), the model accelerates fibrosis progression and increases susceptibility to HCC. Compared to mice receiving CCl_4_ injection alone, animals subjected to both HFD and CCl_4_ exhibit an enhanced fibrotic response and accelerated hepatocarcinogenesis [56].

Although the synergistic effect of HFD feeding and CCl_4_ injection significantly shortens the experimental duration required for MASH progression—making it particularly valuable for investigating fibrogenic mechanisms and evaluating anti-fibrotic therapies—researchers should note that the accelerated fibrosis driven by direct hepatotoxicity may not fully recapitulate the gradual fibrogenesis observed in human disease progression.

#### 3.2.2. Streptozotocin and High-Fat-Induced Mouse Model (Stelic Animal Model, STAM Model)

Streptozotocin (STZ), a cytotoxic compound derived from *Streptomyces achromogenes*, is selectively internalized by pancreatic β-cells via glucose transporter 2 (GLUT2). Administration of STZ initiates an inflammatory cascade mediated by infiltrating macrophages and T lymphocytes, leading to β-cell destruction [57]. The conventional STAM protocol comprises a single subcutaneous injection of 200 μg STZ in 2-day-old *C57BL/6J* mice, followed by HFD feeding at 4 weeks of age to promote HCC. Exposure to STZ induces permanent pancreatic damage, impaired β-cell function, and persistent hyperglycemia in neonatal mice [58]. This model accelerates disease progression, with steatosis, inflammation, and hepatocyte ballooning evident within 6–8 weeks after HFD feeding, followed by progressive fibrosis and development of MASH-associated HCC by week 20 [24]. However, this method has obvious limitations, with a mortality rate exceeding 50%, and cannot recapitulate key metabolic features of human MASLD, such as obesity and IR. Therefore, although the tumor incidence rate of STAM is very high, its lack of characteristic metabolic disorder significantly limits its translational application in studying the metabolic mechanisms of MASH-associated HCC [59].

An alternative protocol involves consecutive intraperitoneal injections of low-dose STZ (40 mg/kg for 5 days) in 7-week-old *C57BL/6J* mice, followed by HFD feeding at 8 weeks of age. This optimized method induces a progressive disease phenotype with distinct temporal stages: steatosis (week 14), MASH (week 20), fibrosis (week 32), advanced fibrosis (week 44), and HCC (week 68). Importantly, this refined model exhibits hepatic transcriptomic profiles that closely resemble those of obese patients with type 2 diabetes (T2D) and MASH-associated HCC [60]. Furthermore, therapeutic intervention with tirzepatide, a dual GIP/GLP-1 receptor agonist, effectively ameliorates MASH pathology, highlighting its utility for the preclinical evaluation of metabolic-targeted therapies [61].

### 3.3. Genetic Engineering Mouse Models

Genetically engineered mouse models (GEMMs) have become indispensable tools for investigating gene function, elucidating molecular mechanisms, and developing novel therapeutic strategies for human diseases. Traditional approaches for generating these models rely on embryonic stem cell (ESC)-based techniques, primarily through gene targeting and gene trapping. Gene targeting utilizes homologous recombination to replace endogenous sequences, whereas gene trapping employs vectors (such as viral vectors or transposons) to achieve random genomic integration [62].

Recent technological advances have significantly expanded the genetic engineering toolkit. Site-specific recombination systems (e.g., Cre/loxP) and programmable nucleases—including zinc-finger nucleases (ZFNs), transcription activator-like effector nucleases (TALENs), and the CRISPR/Cas9 system—have largely supplanted conventional methods. When combined with advanced delivery systems and precise targeting strategies, these technologies enable rapid and accurate manipulation of the mouse genome, accelerating the generation of models that faithfully recapitulate human diseases [63] (Table 3).

#### 3.3.1. Leptin Deficiency (*ob/ob* Mice) Model

Leptin, an adipokine predominantly secreted by white adipose tissue, serves as a central regulator of energy homeostasis [64]. Under physiological conditions, leptin modulates feeding behavior and metabolic functions through a negative feedback loop acting on hypothalamic neurons. The *ob/ob* mouse model harbors a spontaneous mutation in the leptin-encoding *ob* gene, resulting in functional leptin deficiency and severe IR. This deficiency leads to hyperphagia, reduced physical activity, and obesity, accompanied by ectopic lipid deposition in the liver and other non-adipose tissues [65]. Consequently, leptin deficiency promotes hepatocyte lipotoxicity and lipoapoptosis; thus, the *ob/ob* mouse model accurately recapitulates the early metabolic disorder stage of human MASLD, closely mirroring the characteristics of simple hepatic steatosis in humans.

Notably, *ob/ob* mice exhibit significant resistance to liver fibrosis, which presents a disadvantage when studying the progressive fibrotic stages of human MASLD. Several studies support that leptin deficiency reduces extracellular matrix production even under profibrotic stimuli, highlighting the essential role of this hormone in fibrogenesis [66]. Due to the inhibition of spontaneous MASH in *ob/ob* mice, additional dietary challenges—such as high-fat, high-cholesterol diets or methionine-choline-deficient (MCD) diets—or chemical interventions are required to induce this phenotype. For instance, feeding *ob/ob* mice an MCD diet for 4 weeks can trigger the transition from simple steatosis to MASH, characterized by hepatic steatosis, inflammation, and fibrosis [67], closely resembling the phenotype of human MASH. Furthermore, GAN diet intervention can elicit a more severe MASH phenotype with fibrosis in *ob/ob* mice compared to wildtype mice. Importantly, these phenotypes are consistent with the transcriptome of human MASH, indicating the potential value of the GAN diet-induced *ob/ob* mouse model in evaluating novel therapeutic agents for progressive MASH [68].

Despite the utility of dietary interventions in the *ob/ob* mouse model, clinical data indicate that its translational value remains limited. In contrast to *ob/ob* mice, serum leptin levels in human MASLD/MASH patients are not lower than those in healthy controls [69]. This discrepancy suggests that *ob/ob* mice cannot fully recapitulate the hormonal milieu of human MASH, particularly in advanced fibrotic stages. Therefore, careful consideration is required when using this model to mimic the intermediate and advanced stages of human MASLD.

#### 3.3.2. Leptin Receptor Deficiency (*db/db*) Mouse Model

The *db/db* mouse, characterized by a point mutation in the *Lepr* gene leading to leptin receptor deficiency, is a valuable model for studying T2D and associated hepatic complications. These mice exhibit normal or elevated circulating leptin levels but are unable to respond to leptin signaling due to receptor dysfunction. Consequently, they develop a spectrum of metabolic abnormalities, including obesity, IR, hepatic steatosis, and diabetes, making them suitable for preclinical research on MASLD.

Although *db/db* mice share phenotypic similarities with *ob/ob* mice—such as severe obesity and hyperphagia—they display distinct metabolic characteristics. For instance, *db/db* mice exhibit more pronounced adipose tissue inflammation but relatively milder hepatic inflammation. Moreover, *db/db* mice accumulate more subcutaneous fat, whereas *ob/ob* mice show greater epididymal fat deposition [70]. These metabolic disturbances closely mimic the early metabolic disorder stage of human MASLD, characterized by IR, dyslipidemia, and isolated hepatic steatosis.

Similar to other genetic models, *db/db* mice generally do not spontaneously progress to the fibrotic stages of MASLD. Induction of severe MASH, characterized by significant necroinflammation and fibrosis, requires additional insults such as methionine-choline-deficient (MCD) diets, high-fat diets (HFD), and chemical agents [71,72]. Notably, iron overload has been shown to accelerate disease progression in *db/db* mice, promoting the transition from steatosis to steatohepatitis and fibrosis through mechanisms involving oxidative stress, mitochondrial dysfunction, and impaired insulin signaling [73]. The iron-induced phenotype may partially recapitulate the progressive fibrotic stage of human MASH. However, it remains unclear whether *db/db* mice can develop advanced bridging fibrosis.

Collectively, the *db/db* mouse model reliably recapitulates the metabolic features of early-stage human MASLD. When combined with nutritional or chemical challenges, it can simulate key features of MASH with early fibrogenesis. Nevertheless, it fails to fully represent the late stages of human MASH, such as advanced fibrosis and HCC.

#### 3.3.3. The *foz/foz* Mouse Model

The *Alms1* gene encodes a centrosomal protein essential for ciliogenesis and microtubule organization. Loss-of-function mutations in the human *ALMS1* gene cause Alström syndrome, a rare autosomal recessive disorder with multi-organ involvement. The *foz/foz* mouse, which carries a truncating mutation in the *Alms1* gene, spontaneously develops obesity, insulin resistance, and hepatic steatosis, making it a suitable model for studying MASLD.

The most distinctive feature of this model is its high reproducibility and rapid disease onset under dietary challenge. It faithfully recapitulates the entire spectrum of human MASLD progression, from steatosis to MASH, advanced fibrosis, and HCC. Upon initiation of a Western diet, mice develop hepatic steatosis within 1–2 weeks, progress to MASH with inflammation and hepatocellular ballooning by 4 weeks, and exhibit significant (grade 3) fibrosis by week 12. Extending the dietary intervention to 24 weeks results in cirrhosis accompanied by spontaneous HCC and severe extrahepatic complications, such as cardiac dysfunction, thereby mirroring the end-stage manifestations of human disease [74,75].

It is noteworthy that the severity of the fibrotic phenotype is strain-dependent; mice on a *C57BL/6J* background develop more severe liver fibrosis than those on a BALB/c background. Transcriptomic analysis further validates its translational potential, demonstrating that 257 genes—including *Col1a1*, *Lgals3*, and *Spp1*—are similarly dysregulated in the livers of *foz/foz* mice fed a Western diet for 12 weeks and in human MASH [75].

#### 3.3.4. *B6-Alms1-del* Mice Model

To further investigate the pathological consequences of *Alms1* deficiency, we generated the *B6-Alms1-del* model via CRISPR/Cas9-mediated introduction of an 11 bp deletion in exon 8 on a *C57BL/6J* background. Unlike the acute pathology of the *foz/foz* model, *B6*-*Alms1-del* mice exhibit a chronic, progressive disease course that more closely resembles the natural progression of human MASLD. Metabolic disturbances manifest as early as 6 weeks of age, characterized by progressive obesity, hyperglycemia, insulin resistance, and dyslipidemia. By 21 weeks, mice develop significant steatohepatitis. Between 5 and 8 months, the model gradually develops mild hepatic fibrosis while maintaining metabolic stability over an extended period [76]. Furthermore, a high-fat, high-sugar diet exacerbates metabolic dysfunction and liver damage compared to a standard chow diet. This sustained metabolic phenotype distinguishes the *B6-Alms1-del* model from other common metabolic models, such as *ob/ob* or *db/db* mice, which are often limited in long-term fibrosis studies due to transient hyperglycemia or premature mortality.

Therefore, the *B6-Alms1-del* model uniquely fills a critical gap in MASLD research by providing a stable platform with sufficient longevity to observe the complete disease trajectory from metabolic dysfunction to advanced fibrosis. It serves as an invaluable tool for the systematic investigation of MASLD progression, the exploration of stage-specific molecular mechanisms, and the evaluation of long-term therapeutic interventions.

#### 3.3.5. Low-Density Lipoprotein Receptor-Deficient (*Ldlr*^−/−^) Mice and Apolipoprotein E-Deficient (*Apoe*^−/−^) Mice

The *LDLR* (low-density lipoprotein receptor) gene family encodes cell surface proteins critical for the receptor-mediated endocytosis of specific ligands, particularly ApoE. These proteins play a pivotal role in maintaining lipid metabolic homeostasis. Both *Ldlr*^−/−^ and *Apoe*^−/−^ mice are well-established models of spontaneous atherosclerosis. Under specific dietary interventions, such as a Western diet, these mice develop hyperlipidemia, (IR, hepatic steatosis, and inflammation, making them valuable platforms for studying MASLD/MASH [77,78,79].

*Ldlr*^−/−^ mice exhibit diet-accelerated disease progression that faithfully recapitulates key stages of human MASLD. Within 7 days of high-fat, high-cholesterol feeding, they develop visible hepatic steatosis and inflammation. With prolonged HFD feeding, increased uptake of oxidized LDL aggravates liver injury and inflammation. Notably, diet composition significantly influences the severity of MAFLD. Fast food diets can induce advanced bridging fibrosis (F3), whereas HFD exacerbates hyperinsulinemia. Collectively, this model effectively mimics MASH with fibrotic progression [80,81].

Similarly, *Apoe*^−/−^ mice develop spontaneous hyperlipidemia and hypercholesterolemia due to disrupted lipoprotein metabolism. After 7 weeks of HFD feeding, the mice progress from systemic metabolic disorders to steatohepatitis and subsequent fibrosis, corresponding to advanced MASH [82]. Furthermore, high-fat, high-cholesterol diets exacerbate macrovesicular steatosis and inflammation in *Apoe*^−/−^ mice. Nutritional interventions, such as supplementation with flaxseed oil, can attenuate MASLD symptoms and reduce transaminase levels [83].

#### 3.3.6. Models of Human Genetic Risk Variants

The *PNPLA3* rs738409 variant (encoding the I148M protein) is the strongest common genetic risk factor for MASLD and its progression [13]. Researchers have generated precise *Pnpla3* I148M knock-in (KI) mice using homologous recombination techniques, facilitated by CRISPR-Cas9 [84,85]. To observe the slow progression from simple steatosis to MASH and further to advanced fibrosis or hepatocellular carcinoma, these knock-in mice are exposed to long-term (6–12 months) dietary challenges, such as a WD, HCD, or MCD diet [86,87]. This model directly tests the causal role of the variant.

Similarly, knock-in models for other GWAS-identified variants (e.g., *TM6SF2* E167K, *MBOAT7*, *GCKR*) are being developed to dissect their individual and combinatorial effects on liver lipid metabolism and injury [14]. These models move beyond correlation to causation, allowing detailed investigation of how specific human polymorphisms alter cellular pathways—such as lipid droplet remodeling, VLDL secretion, and retinol metabolism—to drive disease.

#### 3.3.7. Other Genetic Modified Mouse Models

In addition to well-established genetically engineered models, several specialized mouse strains offer unique insights into MASLD pathogenesis. Adenosine kinase-deficient (*ADK*^−/−^) mice constitute one such model. ADK regulates intracellular adenosine levels by converting adenosine to AMP in the liver, thereby maintaining adenosine nucleotide homeostasis and supporting S-adenosylmethionine-dependent methylation reactions. *ADK*^−/−^ mice develop marked hepatocellular ballooning and steatosis as early as four days after birth, succumbing to fatal liver failure within two weeks [88]. This model simulates rapidly progressive neonatal fatty liver disease, highlighting the critical role of adenylate metabolism in regulating hepatic lipids during the postnatal period. Recent studies further demonstrate that hepatocyte-specific ADK overexpression exacerbates adiposity, hepatic steatosis, and inflammation, whereas ADK knockdown attenuates lipid accumulation. These findings confirm that hepatocyte ADK promotes MASLD pathogenesis by suppressing fatty acid oxidation and inducing inflammation, indicating its potential as a therapeutic target for obesity and metabolic liver diseases [89].

The scavenger receptor CD36 is another key regulator of MASLD progression. As a membrane glycoprotein expressed on hepatocytes and macrophages, CD36 facilitates long-chain fatty acid uptake and triglyceride storage. Bone marrow transplantation experiments show that CD36 deficiency in *Ldlr*^−/−^ mice fed a HFD reduces hepatic inflammatory cell infiltration and decreases cholesterol accumulation in KCs [90]. Conversely, CD3 deluxe overexpression increases free fatty acid uptake and promotes MASLD progression through multiple mechanisms, including oxidative stress, impaired autophagy, lipotoxicity, and inflammation [91]. In human MASH, CD36 is significantly overexpressed and hyper-palmitoylated in hepatocytes. Importantly, inhibiting CD36 palmitoylation attenuates murine MASH progression [92], underscoring the therapeutic potential of targeting CD36 post-translational modifications in metabolic liver disease.

### 3.4. Recent Advances in Organoid Models for MASLD

Organoid models have emerged as a pivotal platform in MASLD research, offering the capacity to recapitulate disease mechanisms while providing robust support for therapeutic discovery [93].

Through single-cell transcriptomics, researchers have stratified MASLD organoid models and established a framework aligning in vitro systems with clinical disease progression. This advancement lays the foundation for benchmarking organoid injury models and conducting in-depth investigations into steatohepatitis, fibrosis, and hepatic stellate cell dynamics [94]. On this basis, in vitro human organoid models specifically tailored to MASLD have been developed, further improving the accuracy of disease representation [95]. Concurrently, various ex vivo platforms—including liver-on-a-chip systems and organoid-derived tissue-like constructs—have demonstrated significant value in reducing reliance on in vivo models while accelerating drug discovery and biomarker identification [96]. In the realm of cellular interaction studies, three-dimensional coculture liver organoids have been shown to recapitulate inter-tissue communication during MASLD progression, indicating potential applications in therapeutic transplantation [97]. Furthermore, dynamic three-dimensional coculture systems can simulate distinct disease stages, providing robust platforms for mechanistic investigation and therapeutic screening [98]. Organoid systems have also been widely utilized for therapeutic evaluation. In tissue-engineered liver organoids, naringin has been demonstrated to alleviate MASLD-associated liver injury by inducing autophagy through the MTOR-ULK1 pathway [99]. Additionally, a convective transport-enhanced multi-organoid device has been developed to overcome the limitations of passive diffusion, enabling a more faithful recapitulation of tissue-specific functions and physiological gradients within the context of obesity [100]. Notably, the successful construction of multi-cellular human liver organoids incorporating hepatocytes, macrophages, and stellate cells has enabled a more accurate recapitulation of MASLD pathology and xenobiotic metabolism, underscoring the critical role of cellular complexity in modeling disease mechanisms and drug responses [101].

Collectively, these advances highlight the substantial potential of MASLD organoid models in mirroring disease progression, elucidating underlying mechanisms, and serving as preclinical platforms for therapeutic evaluation. With the continued integration of single-cell transcriptomics, multi-cellular architectures, and dynamic culture conditions, these models are being progressively refined, holding promise for the development of more physiologically relevant and clinically translatable tools for MASLD research.

### 3.5. Animal Models Correlating Immune Cells for the Development of MASLD/MASH

Current research on immune-induced MASLD models highlights the essential contributions of both innate and adaptive immunity to disease progression and therapeutic development. Key components of the innate immune response—including the TLR4 signaling axis in KCs, the NLRP3 inflammasome, and related molecules such as MyD88, iNOS, and HMGB1—drive hepatic inflammation, steatosis, and fibrosis by regulating pro-inflammatory cytokine release; correspondingly, gene knockout can alleviate MASLD-related pathological alterations [102,103]. The role of adaptive immunity is more complex: while T cells are implicated in the progression from MASLD to HCC [104], regulatory T cells appear dispensable for early MASLD development, suggesting a dominant role of innate immunity in the initial stages [105]. Furthermore, targeting immune-related molecular pathways offers viable therapeutic strategies. Specifically, modulating Mitochondrial antiviral signaling protein (MAVS)-mediated signaling [106], silencing the lipid metabolism regulator PLIN2 [107], applying liver-specific antisense oligonucleotides against MDM2 [108], and regulating C6ORF120-related PPAR pathways can effectively ameliorate hepatic steatosis, inflammation, and fibrosis in preclinical MASLD models [109].

## 4. Conclusions and Perspective

MASLD affects more than one-quarter of the global population and exhibits strong associations with metabolic disorders. Its inflammatory subtype, MASH, has emerged as a growing health burden and a leading cause of morbidity, mortality, and liver transplantation worldwide [110]. The significant heterogeneity in MASH phenotypes, driven by racial, genetic, and environmental factors, highlights the necessity for personalized treatment strategies [111,112]. This demand has accelerated the development of various preclinical models and driven clinical trials to validate intervention targets.

The continuous evolution of MASLD treatment strategies has driven the refinement of animal models targeting specific disease symptoms. Several studies have attempted to induce MASLD phenotypes in human hepatocyte chimeric mice through specific dietary regimens, such as HFD or MCD diet. By transplanting human hepatocytes into immunodeficient mice, this model reconstructs human-specific metabolic and transport pathways, significantly improving the predictive accuracy for pharmacokinetics, drug interactions, and hepatotoxicity [113]. Beyond well-established models, pharmaceutical development has introduced novel animal models, such as *PPARα^−/−^* mice, to serve clinical translation research through dietary, chemical, and genetic interventions [114]. Drug discovery has expanded beyond traditional small molecules and monoclonal antibodies into new fields such as RNA-based therapy, gene therapy, and microbiome-targeted interventions. For instance, *Bifidobacterium pseudolongum* has been demonstrated to attenuate the progression of MASLD to HCC [115]. Meanwhile, metabolomic and lipidomic analyses aid in discovering promising diagnostic biomarkers, such as specific amino acids, fatty acids, triglycerides, phospholipids, and bile acids [116]. The emergence of these new therapeutic strategies and diagnostic biomarkers has raised higher requirements for the validation capabilities of preclinical models.

In this context, technological advancements facilitate the more effective recapitulation of complex disease pathological manifestations in preclinical models. Recently, traditional two-dimensional cultures are being replaced by three-dimensional bioscaffold systems, which better mimic the pathophysiological status of adipocyte metabolism [117]. Additionally, under perfusion 3D culture conditions, liver organoids derived from human-induced pluripotent stem cells (hiPSCs) can reproduce key MASLD features following prolonged free fatty acid stimulation [118]. Microphysiological systems (MPS) incorporating dynamic fluid flow address the critical shortcomings of static cultures by simulating hemodynamic shear stress and improving nutrient/waste exchange [119,120,121,122]. These systems now support the study of lipid droplet dynamics and multi-organ interactions and have been validated on a modular chip by simulating the cardiovascular-kidney-metabolic syndrome [123].

Nevertheless, these new models still present significant limitations, primarily reflected in their insufficient capacity to fully recapitulate the chronic progression and multisystem interactions underlying MASLD. First, current humanized models predominantly focus on either hepatic or immune system humanization, making it difficult to comprehensively simulate the multisystem interactions (e.g., the gut–liver axis, adipose tissue–liver axis) that underpin MASLD. Moreover, most existing models lack a functional immune system and are thus unable to fully recapitulate the complex immune-inflammatory processes involved in the progression from simple steatosis to MASH. Second, although organ-on-a-chip technology offers advantages in simulating hemodynamic shear stress and nutrient/waste exchange, its culture duration is typically constrained to approximately four weeks, making it difficult to model the chronic progression of MASLD from simple steatosis to advanced fibrosis. Meanwhile, this technology continues to face technical challenges in incorporating dynamic immune components, such as macrophages and T cells. Finally, key technical parameters of organoid systems, such as cell density and flow rate, currently lack standardized protocols, resulting in poor reproducibility across models and impeding cross-laboratory comparison and validation of data. This limitation restricts their broad application in early-stage drug screening and development.

To overcome these limitations, future research should focus on the construction of multisystem-integrated models, including: multi-organ chip integration, which connects key organ chips such as the liver, gut, and adipose tissue to faithfully recapitulate the systems biology features of MASLD; dynamic immune microenvironment reconstruction, which develops sustainably circulating immune cell co-culture systems to precisely simulate the spatiotemporal evolution of immune-inflammatory responses; and deep learning-driven parameter standardization, which utilizes artificial intelligence to dissect the biological characteristics of organoids and establish unified quality control standards, thereby enhancing model predictability and reproducibility. In conclusion, although current MASLD models have achieved important breakthroughs in mechanistic dissection and drug discovery, realizing true precision medicine applications will require comprehensive technological iteration in systemic integration and standardization to construct next-generation preclinical models that more closely resemble human pathophysiological states, thereby accelerating the clinical translation of precision therapeutic strategies for MASLD.

## Figures and Tables

**Table 1:** Diet-induced MASLD mouse model. The table summarizes the phenotypic characteristics, induction time, and key features of various dietary mouse models used in the study of MASLD and its progression to MASH. Key parameters include the presence of obesity, nutritional composition, insulin resistance, steatosis, inflammation/ER stress, fibrosis, HCC, and the typical modeling duration. Y, yes; N, no; MCD, methionine-choline deficient; HFD, high-fat diet; HFHC, high-fat high-cholesterol; GAN, Gubra-Amylin NASH; ALOS, American Lifestyle-Induced Obesity Syndrome.

Models	Nutritional Composition	Obesity	Insulin Resistance	Steatosis	Inflammation/ ER Stress	Fibrosis	HCC	Weekly Age of Application	Features
Methionine- and choline-deficient (MCD) mouse models	40% sucrose and 10% fat, lacking methionine and choline	N	N	Y	Y	Y	Y	MASLD (Severe steatosis, hepatocyte ballooning, inflammation): 2–4 weeks	Significant differences from human metabolic profiles, which cannot be used to study early stage of MASLD, and can be used to study late stage.
								MASH/Fibrosis: 8 weeks	
								HCC: 12–18 weeks	
High-fat diet (HFD) mouse models	45% fat	Y	Y	Y	Y	delayed induction	N	Early-stage MASLD (simple steatosis): 6–12 weeks	The model is similar to human disease development, but it is time-consuming to reproduce MAFSLD, and difficult to develop fibrosis and HCC.
	45% or 60% fat diets/71% fat, 11% carbohydrate and 18% protein							MASH: 16–24 weeks	
High-cholesterol diet (HCD) mouse models	1% cholesterol and 4% fat	Y	Y	Y	N	N	N	—	The model can cause more severe liver injury and induce fibrosis, addressing the shortcoming of high-fat diet models.
Choline-deficient L-amino-defined diet (CDAA)-induced mouse model	0.1% methionine and 45% fat	N	N	Y	Y	Y	Y	MASH: 3–6 weeks	No weight gain and insulin, it is inconsistent with human disease metabolism; however, the combination of high-fat diet-induced animal models can better simulate the human MASLD process, effectively compensating for the shortcomings of the MCD model.
								Fibrosis/HCC: 12–36 weeks	
High-fat and high-cholesterol (HFHC) diet-induced mouse model	>15% fat, 0.2–1% cholesterol, often supplemented with sucrose and cholate	Y	Y	Y	Y	Y	N	Early MASH: 8–12 weeks	The HFHC diet-induced model is simple and reliable, and its phenotype is more similar to that of humans, but it is insulin intolerant, and excess choline may increase the risk of fibrosis, so the model is usually used in obesity-susceptible mice.
	30% fat, 1.25% cholesterol, and 0.5% bile							MASH/Fibrosis: 16–24 weeks	
GAN DIO-MASH diet-induced mouse model	40% saturated fat, 22% fructose, and 2% cholesterol	Y	Y	Y	Y	Y	N	MASH/Fibrosis: 38 weeks	The GAN DIO-MASH mouse model confirm significant clinical relevance in histopathological features, gene transcription patterns and metabolic characterization of the human disease, highlighting the potential of the GAN DIOMASH mouse model for the discovery of MASH therapeutic targets and the evaluation of novel drug therapies.
“American Lifestyle-Induced Obesity Syndrome” (ALIOS) mouse model	high-fat diet (containing 30% trans fatty acids) and high fructose corn syrup (containing 55% fructose and 45% sucrose) in drinking water	Y	Y	Y	Y	Y	Y	MASLD: 8–16 weeks	The ALIOS mouse encapsulates many of the clinical features of MASLD and therefore represents a robust and reproducible model for studying the pathogenesis of MASLD and its progression.
								MASH/HCC: 6–12 months	

**Table 2:** Chemical induction and composite models.

Models	Dosages	Obesity	Insulin Resistance	Steatosis	Inflammation/ ER Stress	Fibrosis	HCC	Weekly Age of Application	Features
High-fat diet plus carbon tetrachloride (CCl4) mouse model	HFD feeding, low-dose CCL4 (0.5–0.8 mL/kg) administration	Y	Y	Y	Y	Y	Y	MASLD: 2–4 weeks	As a classical model, it can effectively save time and resources, but its pathogenesis as well as disease characterization is not consistent with humans, and it can be used in combination with dietary models.
								MASH/Fibrosis: 8–12 weeks	
								HCC: 16–24 weeks	
Streptozotocin and high-fat-induced mouse model (STAM model)	A single subcutaneous injection of 200 μg STZ in 2-day-old *C57BL/6J* mice and feeding HFD at 4 weeks of age	N	N	Y	Y	Y	Y	Conventional STAM: MASH at 6–8 weeks, HCC by 20 weeks(rapid but high mortality).	This model has a short modeling time and a high tumor incidence; however, the mice lose weight and there is a gender difference in tumor production, which is also a limitation of this model.
	Low-dose STZ (40 mg/kg for 5 days) in 7-week-old *C57BL/6J* mice, followed by HFD feeding at 8 weeks of age							Optimized STAM: Well-defined pathological stages—steatosis (14 weeks), MASH (20 weeks), fibrosis (32 weeks), advanced fibrosis (44 weeks), HCC(68 weeks).	

**Table 3:** Genetic engineering models.

Models	Obesity	Insulin Resistance	Steatosis	Inflammation/ ER Stress	Fibrosis	HCC	Weekly Age of Application	Features
Leptin deficiency (*ob/ob* mice) model	Y	Y	Y	N (depends on external stimuli such as diet)	N (resistance to fibrosis)	N	—	Similar to human disease manifestations except for the inability to develop fibrosis and hepatocellular carcinoma, which can be applied to study the early stage of MASH.
Leptin Receptor Deficiency (*db/db*) Mouse Model	Y	Y	Y	N (depends on external stimuli such as diet)	N (depends on external stimuli such as diet)	N	—	Similar to *ob/ob* model but can combined with dietary models for fibrogenesis.
The *foz/foz* mouse model	Y	Y	Y	Y	Y	Y	Hepatic steatosis: 1–2 weeks	Provides a stable and highly relevant model for the study of MASH and MASH-associated HCC, demonstrating that liver transcriptomic and histologic features of *foz/foz* MASH mice are highly consistent with human MASH.
							MASH: 4 weeks	
							Significant fibrosis: 12 weeks	
							Cirrhosis/HCC: 24 weeks	
Low-density lipoprotein receptor-deficient (*Ldlr*^−/−^) mice and apolipoprotein E-deficient (*Apoe*^−/−^) mice	Y	Y	Y	Y	Y	N	—	*Ldlr*^−/−^ and *Apoe*^−/−^ mice are hyperlipidemia-driven models that rapidly recapitulate key features of MASLD/MASH under dietary challenge, offering unique insights into the role of dysregulated lipoprotein metabolism and cholesterol in liver disease progression.
*B6-Alms1-del* Mice	Y	Y	Y	N	Y	N	Hepatic steatosis: 6–21 weeks of age	*B6-Alms1-del* is a chronic progressive MASLD model that enables long-term observation of the natural transition from metabolic dysregulation to early fibrosis due to extended animal survival.
							Mild hepatic fibrosis: 5–8 months of age	

## Data Availability

No new data were created or analyzed in this study. Data sharing is not applicable to this article.
